# Allopatric Speciation within a Cryptic Species Complex of Australasian Octopuses

**DOI:** 10.1371/journal.pone.0098982

**Published:** 2014-06-25

**Authors:** Michael D. Amor, Mark D. Norman, Hayley E. Cameron, Jan M. Strugnell

**Affiliations:** 1 Genetics Department, La Trobe Institute for Molecular Science, La Trobe University, Bundoora, Victoria, Australia; 2 Science Department, Museum Victoria, Carlton, Victoria, Australia; 3 School of Biological Sciences, Monash University, Clayton, Victoria, Australia; College of Charleston, United States of America

## Abstract

Despite extensive revisions over recent decades, the taxonomy of benthic octopuses (Family Octopodidae) remains in a considerable flux. Among groups of unresolved status is a species complex of morphologically similar shallow-water octopods from subtropical Australasia, including: Allopatric populations of *Octopus tetricus* on the eastern and western coasts of Australia, of which the Western Australian form is speculated to be a distinct or sub-species; and *Octopus gibbsi* from New Zealand, a proposed synonym of Australian forms. This study employed a combination of molecular and morphological techniques to resolve the taxonomic status of the ‘tetricus complex’. Phylogenetic analyses (based on five mitochondrial genes: *12S* rRNA, *16S* rRNA, *COI, COIII and Cytb*) and Generalised Mixed Yule Coalescent (GMYC) analysis (based on *COI*, *COIII* and *Cytb*) distinguished eastern and Western Australian *O. tetricus* as distinct species, while *O. gibbsi* was found to be synonymous with the east Australian form (BS = >97, PP = 1; GMYC p = 0.01). Discrete morphological differences in mature male octopuses (based on sixteen morphological traits) provided further evidence of cryptic speciation between east (including New Zealand) and west coast populations; although females proved less useful in morphological distinction among members of the tetricus complex. In addition, phylogenetic analyses suggested populations of octopuses currently treated under the name *Octopus vulgaris* are paraphyletic; providing evidence of cryptic speciation among global populations of *O. vulgaris,* the most commercially valuable octopus species worldwide.

## Introduction

Taxonomy within the benthic octopuses (Family Octopodidae) continues to be a source of confusion and controversy and despite extensive revisions in recent decades, the true taxonomy of this family remains unresolved [1], [2], [3]. The most widely studied and economically significant group of cephalopods worldwide is the ‘*Octopus vulgaris* group’ of octopods. The type species of this group is the common octopus, *Octopus vulgaris* Cuvier, 1797. *Octopus vulgaris* alone accounts for >50% of the world's total octopod fisheries catch, exceeding 380,000 tonnes and has an international export value of >US$1 billion [4]. The *Octopus vulgaris* species group is comprised of tropical, sub-tropical and temperate species from the Americas, Europe, Africa, Asia and Australasia. Members of this group are large muscular octopuses that display similar morphological and behavioural traits as well as occupying similar ecological niches.

Within the subtropical waters of Australasia there is a group of morphologically, behaviourally and functionally similar *Octopus* species, closely related to *Octopus vulgaris* [3], [5]. These species, currently treated under the names *Octopus tetricus* on the east and west coasts of Australia and *O. gibbsi* in New Zealand, have been suggested to be a species complex; the taxonomy of which remains unresolved [3]. We treat these taxa collectively herein as the ‘tetricus complex’, after the first formally described species within this group, *Octopus tetricus* Gould, 1852; the common Sydney octopus.

**Figure 1:**
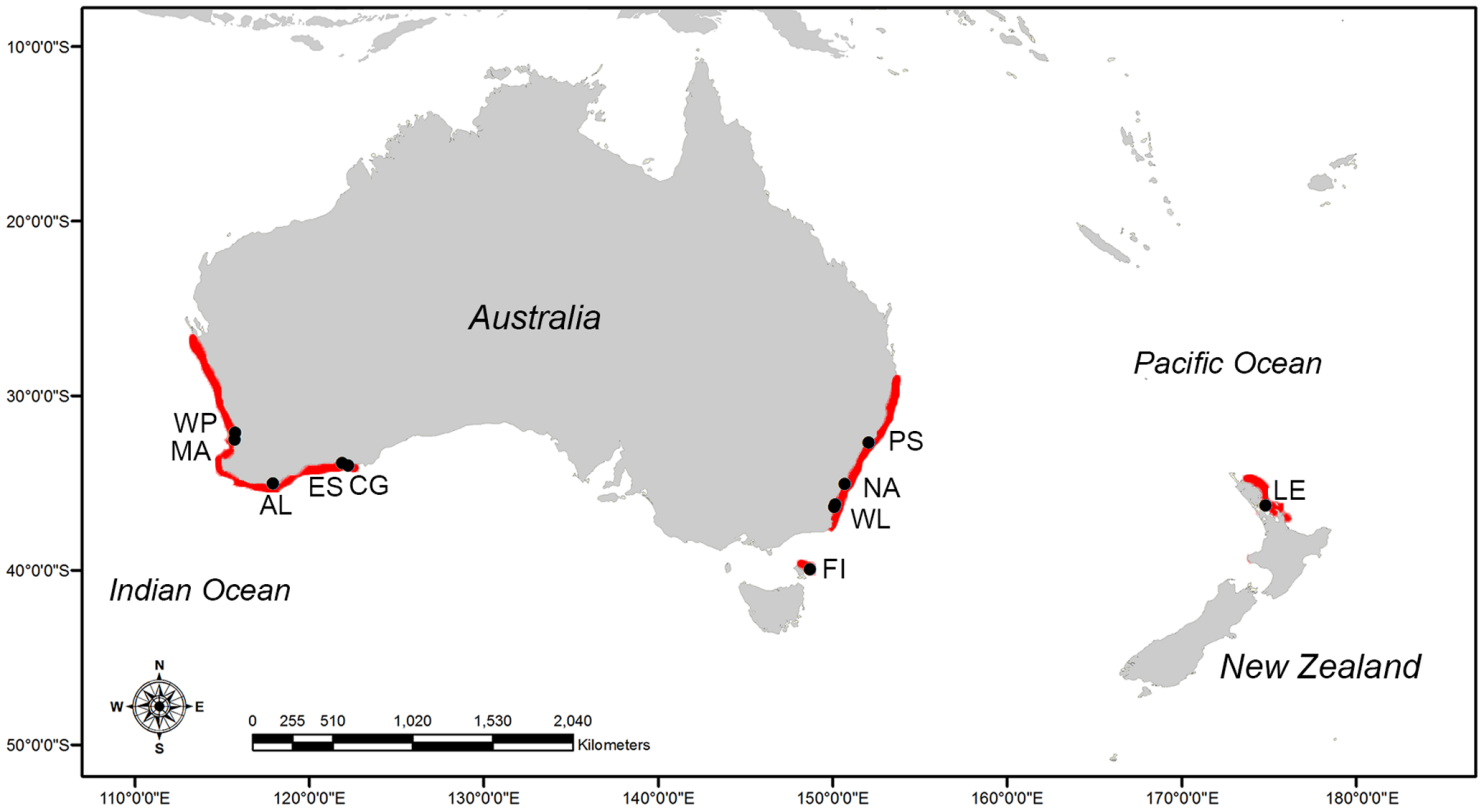
Known distributions (shown in red) and sample locations (shown in black) for *Octopus tetricus*, (east Australia), *O.* cf. *tetricus* (Western Australia) and *O. gibbsi* (New Zealand). Location acronyms: WP =  Woodman's Point, MA =  Mandurah, AL =  Albany, ES =  Esperance, CG =  Cape Le Grand, FI =  Flinders Island, Tasmania, WL =  Wallaga Lake, NA =  Narooma, PS =  Port Stephens, LE =  Leigh, New Zealand.

The tetricus complex comprises three geographically distinct member taxa (Figure 1). *Octopus tetricus* was originally described from New South Wales and occurs along the east Australian coastline, ranging from Eden in southern New South Wales to Moreton Bay in southern Queensland [6]. *Octopus tetricus* comprises a major portion of the small-scale commercial octopod fisheries landings in New South Wales [7], and is also often caught as by-catch in prawn and finfish trawls [8]. Recently *O. tetricus* has been reported in Tasmania, significantly south of its previous known range [9] although this has not been verified by molecular data.

A second taxon, known as the common Perth octopus, occurs in Western Australia from Esperance to Shark Bay. This population has extensively been treated under the name *Octopus tetricus* [10], [11], [12], [13], [14] due to close similarities in morphological, behavioural and functional attributes between east and west coast forms. More recently however, the common Perth octopus has been treated under the name *O.* cf. *tetricus*; a reflection of the proposal that disjunct east and west populations may be sufficiently isolated and therefore represent sub- or distinct species [2], [15]. Joll [11] estimated that 250 tonnes of *O.* cf. *tetricus* were harvested annually from Western Australian waters, primarily as by-catch from lobster fisheries. *Octopus* cf. *tetricus* often preys upon lobsters caught in craypots, and is considered to negatively impact this economically important fisheries resource.

A third nominal species, *Octopus gibbsi* O'Shea, 1999, was coined to describe a benthic octopus of unknown relation found within the shallow coastal waters off northern New Zealand. Prior to description by O'Shea [16], *O. gibbsi* had been treated under the name *O. tetricus* [17], and more recently the validity of *O. gibbsi* as a distinct species has been questioned [2]. Examination of museum specimens showed strong morphological similarities between *O. gibbsi* and Australian forms, leading to the proposal that *O. gibbsi* is synonymous with *O. tetricus* [2].

A phylogenetic analysis of the sub-family Octopodinae using amino acid sequences from two mitochondrial (*cytochrome oxidase subunit III* and *cytochrome b*) and a single nuclear genetic marker (*elongation factor-1α*) assigned *Octopus tetricus* and *O.* cf. *tetricus* as sister taxa [3]. Analyses of genetic distance (Kimura 2 Parameter) between these two representatives showed 2.0% and 2.6% sequence divergence within each mitochondrial gene fragment respectively. However, only single representatives from both Western Australia and New South Wales were sequenced in this study. Consequently, analyses of Guzik *et al.,* [3] were insufficient to detect the occurrence of speciation between disjunct east and west populations, and no traditional morphological based studies comparing the two populations have been conducted. Furthermore, no molecular work to date has investigated the phylogenetic status of *O. gibbsi*, thus its taxonomy remains unresolved.

This study aims to resolve the taxonomic status and phylogenetic relationships of the *Octopus tetricus* species complex, using a combination of molecular and morphological techniques. Due to the emerging fisheries value and the lack of species-level resolution within the tetricus complex, taxonomic resolution within this group will aid in the management of these marine resources.

## Materials and Methods

All tissue samples and DNA extracts were loaned from existing museum/university collections. Thus, no animals were harmed or killed in conducting this study. All appropriate permissions were obtained from the relevant institutions prior to accessing their collections.

### Molecular analyses

#### Sampling

Tissue samples of the ingroup (*Octopus tetricus* [n = 13], *O.* cf. *tetricus* [n = 17] and *O. gibbsi* [n = 4]) were sourced from collections at Museum Victoria, or provided by researchers associated with The University of Adelaide, the Western Australian Fisheries and Marine Research Laboratories Department and the University of Tasmania (Table S1 in File S1). Tissue samples (as arm or mantle tissue ∼1 cm in length) were taken from individuals collected from the Australian mainland, Flinders Island (Tasmania) and New Zealand (Figure 1). All tissue samples were stored at −20°C in 70–90% ethanol until processing.

#### Sequencing

DNA was extracted from mantle or tentacle tissue using the ‘High Salt Method’ [18]. Partial sequences of five mitochondrial genes were targeted; including*12S ribosomal RNA* (*12S*) [19], *16S ribosomal RNA* (*16S*), and *cytochrome oxidase subunits one* (*COI*) [20], *three* (*COIII*) and cytochrome *b* (*Cytb*) [3]. 25 µL reactions comprised 0.1 µL Taq (*Onetaq, New England Biolabs*), 2.5 µL 10 x buffer (Paq5000™), 2 µL dNTP mix (10 µM, *Bioline*), 0.5 µL forward primer (10 µM), 0.5 µL reverse primer (10 µM), 17.4 µL ddH_2_O and 2 µL DNA (diluted to between 1–5 ng/µL). Reaction conditions are detailed elsewhere [21]. PCR products were sequenced by *Macrogen Inc*, Seoul, Korea. Genetic sequences generated in this study are accessible from GenBank under accession numbers KJ605215-KJ605347.

*Octopus mimus* and *O. oculifer* were selected as outgroup taxa on the basis that they are morphologically very similar to, and the closest known available relatives of the ingroup [2], [5], [22]. Sequences of the outgroup and additional sequences of ingroup taxa from previously published work were downloaded from GenBank (Table S2 in File S1). Multiple sequence alignments were performed using *Geneious* Muscle Alignment feature using the ClustalW default settings [23].

#### Phylogenetic analyses

*jModelTest* v0.1.1 [24] was used to carry out statistical selection of best-fit models of nucleotide substitution on the concatenated alignments and also for the *COI* alignment alone. The appropriate model was selected on the basis of ‘goodness of fit measure’ via the Akaike Information Criterion (AIC) [25].

Maximum likelihood (ML) topologies were constructed using *PhyML* v3.1 [26]. Full heuristic searches were undertaken and model parameter values were treated as unknown and were estimated. Strength of support for internal nodes of ML construction was measured using 1000 bootstrap replicates. Bayesian marginal posterior probabilities were calculated using *MrBayes* v3.2 [27]. Model parameter values were treated as unknown and were estimated. Random starting trees were used and the analysis was run for 15 million generations, sampling the Markov chain every 1000 generations. The program *Tracer* v1.3 [28] was used to ensure Markov chains had reached stationarity, and to determine the correct ‘burn-in’ for the analysis (the number of additional generations that must be discarded before stationarity is reached).

#### Genetic distance

*Molecular Evolutionary Genetic Analysis (MEGA)* v5.2 [29] was used to calculate genetic distances for populations of *Octopus tetricus, O. gibbsi* and *O.* cf. *tetricus* using the Tamura-Nei model [30]. Genetic distance was calculated using *MEGA* default settings (with the exceptions of the model and ‘pairwise deletion of missing data’ option). Mean values ± SE of interspecific and intraspecific variations in number of mutations per site were calculated for the barcoding mitochondrial gene *COI* to allow comparison with published literature.

#### Timing of divergence

Divergence time between clades were calculated based on an estimated rate of evolution of cephalopods; 3.81 substitutions per site per billion years (with 95% highest posterior density around this mean of 2.43–5.24; [31]) within a generalised molecular clock.

#### Coalescent delimitation

Potential species delimitation among *Octopus tetricus, O. gibbsi* and *O.* cf. *tetricus* was investigated using a Generalised Mixed Yule Coalescent (GMYC) model [32] applied to the molecular/phylogenetic data. Partitioned sequence data from the mitochondrial genes *COI*, *COIII* and *Cytb* were prepared into XML files using the software program *BEAUti* v1.7.5 [33]. *12S* and *16S* regions were excluded from the analysis due to low comparable sample representation (see Table S1 in File S1). A coalescent prior and relaxed molecular clock [34] were set as parameters before Bayesian analysis was performed using *BEAST* v1.7.5 [33]. Each analysis was performed independently twice and log/tree files were combined using *LogCombiner* v1.7.5 [33]. The data was then analysed via a single threshold model [35] in the software package *Splits* [36] available in *R* v3.0.1 [37], whereby clades with posterior probability values greater than 0.9 were acknowledged.

### Morphological analyses

Morphological data was obtained from preserved whole specimens sourced from Museum Victoria, Australian Museum (Sydney) and the University of Tasmania. Samples were collected from south west (n = 15) and south east (n = 32) of the Australian mainland (between the years 1980–2007) as well as Flinders Island, Tasmania (n = 11; 2011) (Table S3 in File S1). All specimens had been initially fixed in 10% formalin and transferred to 70–90% ethanol for preservation. Morphological data for *O. gibbsi* (n = 6) was sourced from the published work of O' Shea [16].

Specimens were sexed based on three factors which allowed confident classification: 1) presence of terminal organ in males, 2) presence of hectocotylised arm in males and 3) number of genital glands present within the mantle (1 =  male, 2 =  female) [38]. Maturity in males was determined on the basis of the presence or absence of enlarged suckers (for mature and immature specimens, respectively) [39]. Maturity in females was determined by the state of egg development [40]. All specimens were weighed using digital scales to the nearest 0.1 gram after being removed from ethanol and patted dry with absorbent tissue.

Standard morphological characters were measured following Norman and Sweeney [41] (Table 1). Dorsal mantle length (MLd), mantle width (MW), head width (HW), arm width (AW), and the greatest non-enlarged sucker diameter (SDn) were recorded using digital callipers to the nearest 0.1 mm. In males, the greatest enlarged sucker diameter (SDe), the length of hectocotylised arm components (i.e. ligula [LL] and calamus [CL]) and terminal organ length (TOL); following dissection of the mantle, were also measured using digital callipers to the nearest 0.1 mm. For all specimens, third right (ALR3) and third left (ALL3) arm lengths were measured from arm tip to the beak opening using non-stretch string to the nearest 1 mm. The numbers of suckers occurring on the third right (SCR3) and third left (SCL3) arms were counted with the aid of a dissecting microscope. In cases where damage to an arm was perceived to inhibit growth, suckers appeared damaged, or arm regeneration was evident, arm length and sucker counts were not recorded. Where sucker and arm damage was minor, and sucker scars or remnants were visible, suckers and arm lengths were recorded. All missing values for individual traits were replaced with the global mean of that trait across the whole dataset.

**Table 1:** Description of morphological measurements recorded.

Abbreviations	Description
MLd	Dorsal mantle length
MW	Greatest width of mantle
HW	Greatest width of head at the level of eyes
AW	Width of stoutest arm
SDn	Diameter of largest non-enlarged sucker on any arm
WD	Measurement of deepest web sector, from beak to midpoint of sector
ALL3/R3	Length from beak to tip of third left/right arm
SDeL2/R2*	Largest enlarged sucker diameter on the second left/right arm
SDeL3/R3*	Largest enlarged sucker diameter on the third left/right arm
SCL3/R3	Entire number of suckers along intact third left/right arm
LL*	Length from distal most sucker to tip of hectocotylised arm
CL*	Length from distal most sucker to tip of calamus
TOL*	Length of male terminal organ

* Denotes morphological trait only recorded for male octopuses.

All morphological analyses were performed using *Systat v13* [42]. Differences in morphological traits between tetricus complex taxa were investigated using a multivariate General Linear Model (GLM), in which location was treated as a fixed factor, morphological counts were all treated as dependent variables and MLd was entered as a co-variate [43]. Inclusion of MLd as a co-variate controlled for the effect of body size, and therefore allowed investigation of size free shape variation in morphological traits. MLd was considered an appropriate proxy for an individual's body size as it was found to be highly correlated with body mass (R^2^ = 0.8467, data not shown), is more often provided in the literature compared to total body length, and is a standardized measurement when compared to body weight (which can be obtained from fresh or preserved specimens) [44]. The presence or absence of an interaction between locations and MLd was investigated via GLM. A non-significant or weak significant result indicated individuals across all locations were of a similar size class and were therefore comparable.

Males and females were analysed separately to allow the inclusion of male reproductive organs in morphological analyses. Mean scaling was performed on all dependant variables prior to analyses as per Berner [43] using the software package *R* v3.0.1 [37]. The co-variate (MLd) was either log transformed (male) or mean scaled (female) to conform with homogeneity of variance and linearity. Only a single female of appropriate size class/maturity was available from New Zealand, which was excluded from female morphological analyses.

Following multivariate GLM analyses on each of the sexes, principle component (PC) loadings were calculated for each individual by multiplying the mean scaled raw data of each trait by the canonical loading of that trait (supplied by the GLM output) and summing the products for all traits [45]. Principle components were then plotted for visualisation and canonical correlations used to calculate the eigenvalues and proportion of variance explained by each PC (Tables S4-S12 in File S1).

The importance of each morphological character in delineation between tetricus complex taxa was further investigated by Roy-Bargman step-down analysis [46], which has the advantage of retaining information on correlations between multivariate variables compared with univariate F-tests. Following a significant result from GLM analysis morphological traits were ranked in theoretical order of importance by multiplying the first and second canonical loadings (CL1 and CL2) for each trait by the total variance explained by PC1 and PC2, respectively. The resulting values were added together, and traits displaying the highest joint CL were ranked as having the highest priority. Each trait was then investigated sequentially in order of descending ‘importance’ via regression analyses; in which location was a categorical predictor and MLd a co-variate (for size-correction) for all analyses. Higher priority traits were added as co-variates in each successive analysis. Tukey's *post-hoc* tests were performed for each significant step-down analysis to determine differences in morphological traits among locations. Step-down analysis was continued until tests yielded an insignificant effect. Probability values were adjusted via the Bonferroni correction method to account for multiple testing.

To further explore classification of tetricus populations into taxonomic groups, Discriminant Function Analysis (DFA) was performed. As DFA cannot incorporate co-variates, analyses were conducted on calculated principle component loadings for each sex. Principle components were used for DFAs as they were calculated from the original multivariate GLM, and were therefore size corrected. In addition, PCs are composite variables calculated for each individual, and consequently encompass any correlations between morphological traits [45]. For all DFAs, Jackknifed correlation matrices were used as they are considered a more reliable estimator of group membership assignment [47].

## Results

### Molecular analyses

#### Phylogenetic analyses

**Figure 2:**
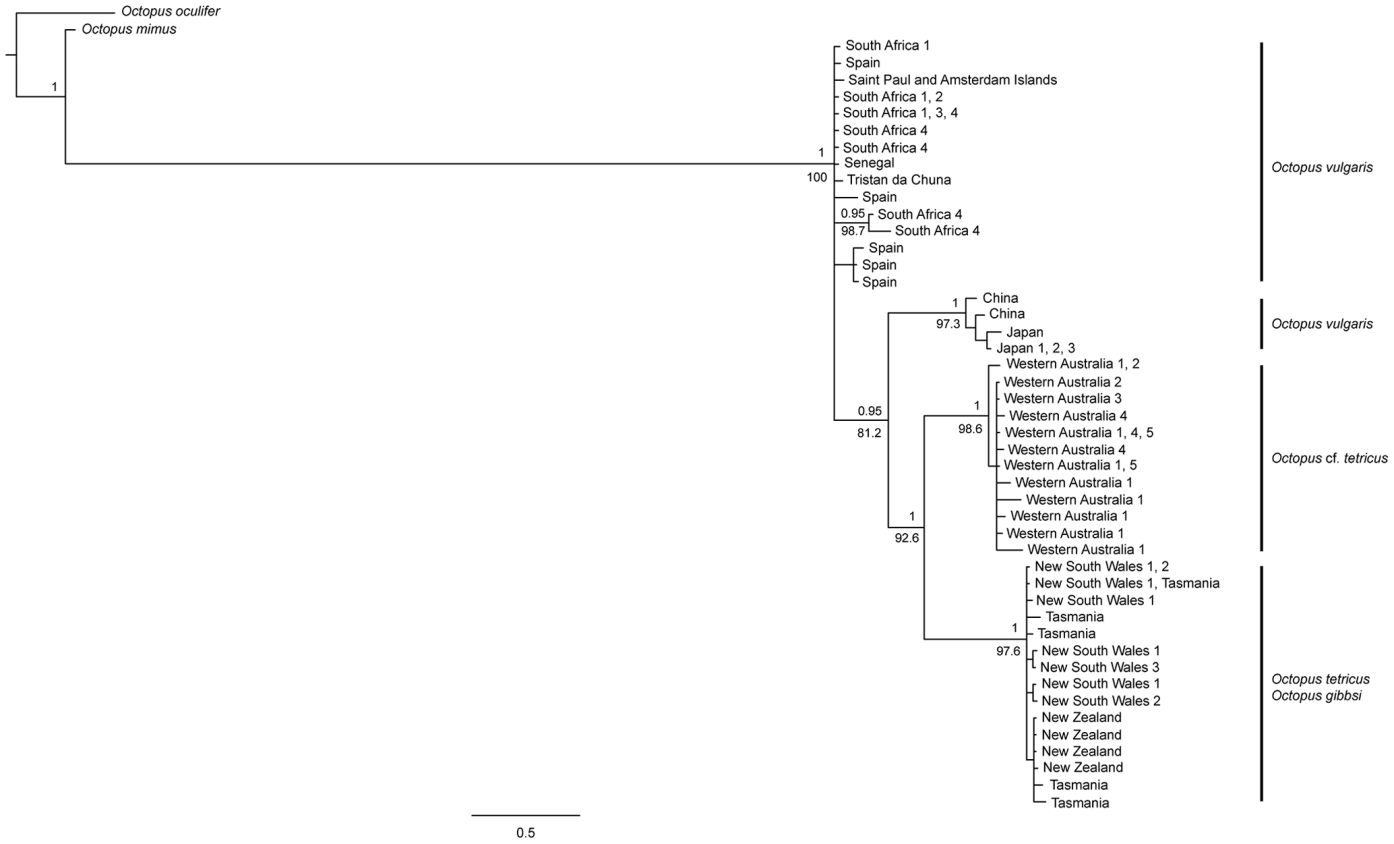
Bayesian topology depicting the phylogenetic relationships among five currently accepted species of Octopoda. Analyses are based on five combined partial mitochondrial genes (*12s* rRNA, *16s* rRNA, *COI*, *COIII* and *Cytb*) showing bootstrap values ≥ 50 below the node and posterior probability values ≥ 0.7 above the node. Outgroup is comprised of *Octopus oculifer* and *O. mimus*. Node labels reflect locations represented by individuals contributing to node (Western Australia, 1 =  Mandurah, 2 =  Woodman's Point, 3 =  Albany, 4 =  Cape Le Grand, 5 =  Esperance; East Australia, 1 =  Wallaga Lake, 2 =  Port Stephens, 3 =  Narooma; South Africa, 1 =  Port Elizabeth, 2 =  Umhlanga, 3 =  Hout Bay, 4 =  Durban).

The AIC indicated that TrN+G was the preferred evolutionary model for the concatenated alignment and this was utilised within ML and Bayesian phylogenetic analyses. Topologies resulting from ML and Bayesian analyses were identical, recovering a highly supported clade containing *Octopus tetricus* from east Australia and Tasmania, as well as *O. gibbsi* from New Zealand (bootstrap value [BS]  = 97.6, posterior probability [PP] =  1; Figure 2). All individuals collected from Western Australia fell within a highly supported monophyletic clade (BS =  98.6, PP =  1). A sister-taxon relationship was supported between the Western Australian and east coast (east Australia, Tasmania and New Zealand) clades (BS =  92.6, PP =  1).

All *Octopus vulgaris* individuals collected from the waters off Japan and China formed a highly supported monophyletic clade (BS =  97.3, PP =  1). The Japanese and Chinese *O. vulgaris* and the tetricus complex were supported as a monophyletic clade (BS =  81.2, PP =  0.95). This clade fell within a larger clade containing *O. vulgaris* individuals from Spain (type location; Mediterranean Sea), South Africa, St Paul and Amsterdam Islands, thereby rendering the *O. vulgaris* clade to be paraphyletic.

#### Genetic distance

*Octopus gibbsi* was treated as *O. tetricus* in genetic distance calculations on *COI* sequence data based on high support values of phylogenetic analyses previously described. Comparisons of within species (i.e. within *O*. cf. *tetricus* or within *O. tetricus/O. gibbsi*) and between species TrN genetic distance for *O. tetricus* (including *O. gibbsi*) and *O.* cf. *tetricus* showed that mean between species divergence (3.34%) was approximately 17.5 times greater than mean within species divergence (0.19%).

#### Timing of divergence

Based on TrN distances, a date of divergence of ∼3.2–6.9 million years ago (ma) was estimated between *Octopus tetricus* from the east coast of Australia (inclusive of *O. gibbsi*) and *O.* cf. *tetricus* from Western Australia (Table S13 in File S1). Furthermore, the Australian tetricus complex clades and the Japanese/Chinese *O. vulgaris* clade were estimated as being separated by ∼5.4–11.6 million years (Table S14 in File S1).

#### Coalescent delimitation

**Figure 3:**
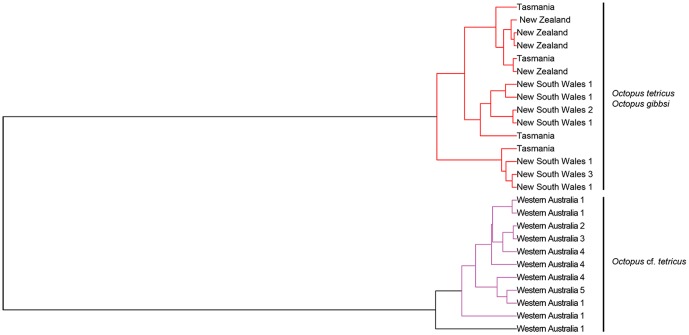
Generalised Mixed Yule Coalescent (GMYC) Bayesian topology depicting the phylogenetic relationships of *Octopus tetricus* (east Australia and Tasmania), *O.* cf. *tetricus* (Western Australia) and *O. gibbsi* (New Zealand). Analysis is based on three concatenated partial mitochondrial genes (*COI*, *COIII* and *Cytb*). Three species clades were supported via GMYC analysis; East Australia and New Zealand (red) and Western Australia (purple and black). Node labels reflect locations represented by individuals contributing to node (Western Australia, 1 = Mandurah, 2 =  Woodman's Point, 3 =  Albany, 4 =  Cape Le Grand, 5 =  Esperance; East Australia, 1 =  Wallaga Lake, 2 =  Port Stephens, 3 =  Narooma).

Two ML clusters and three entities (i.e. species) were supported via GMYC analysis (p = 0.01). All individuals from the east coast of Australia, Tasmania (*Octopus tetricus*) and New Zealand (previously *O. gibbsi*) comprised a single monophyletic clade, whilst the second monophyletic clade was comprised entirely of individuals from Western Australia (Figure 3). A third clade was supported by the GMYC analysis and comprised a single individual from Western Australia, although this clade was paraphyletic, forming a monophyletic clade with other Western Australian individuals.

### Morphological analyses

#### Males

**Figure 4:**
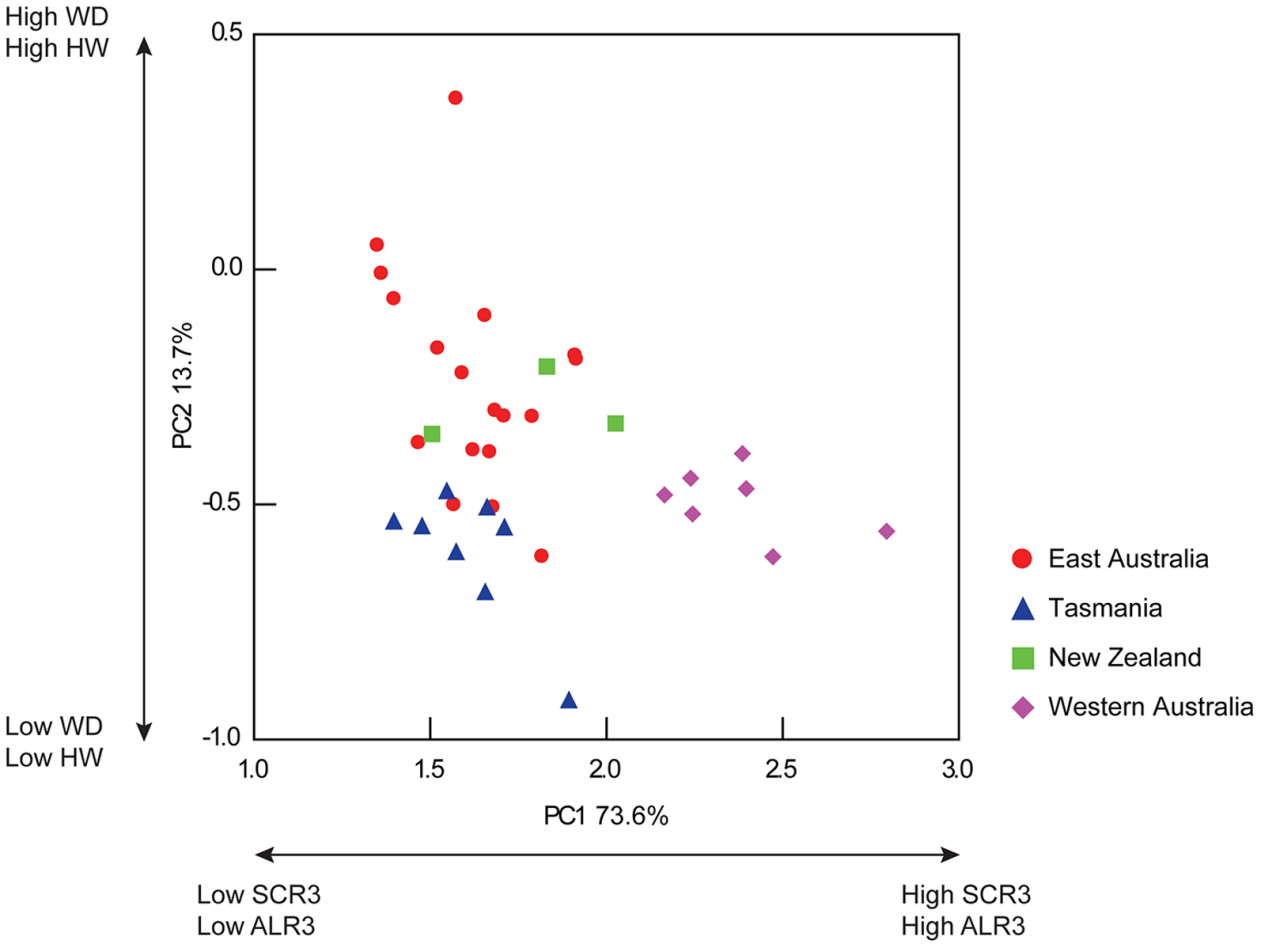
Principal component biplot of male individuals of the tetricus complex. X axis represents PC1 (explaining 73.6% of total variation) and is driven primarily by the SCR3 and ALR3. Y axis represents PC2 (explaining 13.7% of total variation) and is driven primarily by WD and HW.

No strong interaction between the independent variable (coast) and the co-variate (MLd) was recorded (Pillai Trace =  1.937, F = 1.709, *df* = 48,45, p = 0.04), therefore the General Linear Model was run without the interaction. A significant difference was recorded among four coasts for the multivariate model based upon 16 morphological traits (and MLd as co-variate) measured from 36 mature male octopods (Pillai Trace = 2.070, F = 2.503, *df* = 48,54, p = 0.001; Table 2). Visualisation of the male PC biplot showed individuals from the east coast of Australia, Tasmania and New Zealand could not be distinguished from one another, and were characterised by relatively small SCR3 and ALR3 (Figure 4). Western Australian individuals formed a distinct group separate from east coast individuals. Individuals from Western Australia were characterised as having greater SCR3 and ALR3 (PC1) in comparison to individuals from east Australia, Tasmania and New Zealand. No distinctions based upon WD and HW among locations were detected (PC2).

**Table 2:** Summary of mature male morphological measurements taken from preserved museum specimens of *Octopus tetricus* (east Australia and Tasmania), *O.* cf. *tetricus* (Western Australia and *O. gibbsi* (New Zealand).

Catalogue #	Institution	Location	MLd	MW	HW	AW	SDn	WD	ALL3	ALR3	SDeL2	SDeL3	SDeR2	SDeR3	SCL3	SCR3	LL	CL	TOL
C126244	AM	East Australia	69.5	67.2	46.9	23.9	12.3	93	172	193	17.5	16.1	16.6	15.8	162	135	3.6	1.7	11.7
C171685	AM	East Australia	65	67.2	46.9	23.9	12.3	93	279	249	11	11.3	11.3	10.9	220	136	2.9	1.5	9.7
F160334	MV	East Australia	54.3	67.2	46.9	23.9	12.3	93	147	163	17.5	8.2	7.8	7.4	187	143	1.5	0.6	7.5
F77273	MV	East Australia	80.4	49.9	44.6	19.3	9.7	66	311	266	12.1	11.8	12.1	11.5	211	158	3.1	1.2	11.3
F77274	MV	East Australia	86.1	59.6	31.4	15.9	10	86	340	284	13.6	11.9	12.4	13	197	133	3.9	1.7	14
F78281b	AM	East Australia	63.7	64.3	45.2	24.4	12.3	91	227	198	17.5	16.1	16.6	15.8	159	128	2.7	1.1	10.7
F80438	MV	East Australia	89.6	58.7	43.1	24	9.8	99	307	285	17.5	16.1	16.6	15.8	242	153	3.7	1.7	12.9
F80439	MV	East Australia	69.9	55.3	37.8	16.2	9.7	63	298	242	12.7	11.4	10.9	10.7	234	154	3	1.5	10.8
F80440	MV	East Australia	110	66.6	40.9	21.3	12.1	101	435	370	14.7	14.9	14.6	15	190	144	4.6	2.3	15.9
F80445	MV	East Australia	80.1	53.5	41.7	23.5	11.7	73	289	287	18.5	17.5	17.3	16.4	217	121	4.1	1.3	13.1
F200319	MV	East Australia	93.6	55.2	48.7	30.1	13.6	78	365	333	17.6	16.5	17	16.1	217	153	3.5	1.5	15.1
F200324	MV	East Australia	86.7	59.5	44.1	25.5	12.2	88	289	307	18.5	17.8	19.6	17.2	217	138	3	1.3	14.7
F200323	MV	East Australia	85.6	51.8	39	20.5	12.1	74	289	260	14.9	14.5	16.2	14.2	217	139	2.6	0.7	12.8
F200321	MV	East Australia	61.7	64.3	45.2	24.4	12.3	91	211	181	17.5	16.1	16.6	15.8	201	127	3	1.5	9.4
F182058	MV	East Australia	121	88.1	65.5	40	17.3	114	289	426	25.7	24.7	24.7	25.7	217	143	4.2	2.1	19.7
F182057	MV	East Australia	113.7	100.5	64.3	35.4	16.5	113	289	390	22.4	22.6	24.7	21.7	217	150	4.8	2.1	16
F200317	MV	East Australia	122.4	80.6	52.9	28.6	16.4	142	399	456	31.9	26.8	30.2	29.1	142	142	5.2	2.3	14.6
F200318b	MV	East Australia	84.4	57.1	34.1	17.4	9.6	81	345	292	14.1	16.1	14.4	13.1	220	156	3.9	1.3	15.5
F180706	MV	Tasmania	113.2	82.7	58.7	22.9	14	82	470	365	18.7	19.5	23	19.7	195	136	4.5	2	18.1
F180698	MV	Tasmania	101	70.9	37.5	19.5	10.9	72	525	287	17.6	16.5	17	16.1	217	140	3.4	1.8	15
F180707	MV	Tasmania	134.9	71	52.5	29.2	16.3	81	580	468	21.1	25.6	23	22.3	224	146	5.7	2.8	17.6
F180699	MV	Tasmania	90.8	54	34.6	17.7	11.7	81	315	301	17.6	16.5	17	16.1	203	143	3.6	1.5	15.6
F180700	MV	Tasmania	110.9	67.8	37.5	22	11.1	86	408	338	17.6	16.5	17	16.1	212	139	3.8	1.7	14.6
F180697	MV	Tasmania	100.8	56.2	30.2	17.2	9.9	86	403	360	17.6	16.5	17	16.1	218	146	3.4	2.1	16.7
F180696	MV	Tasmania	92.5	56.8	40.7	16.8	9.9	61	413	251	17.6	16.5	17	16.1	201	132	3.7	1.6	12.3
F180702	MV	Tasmania	85	58.4	34.4	16.1	9.2	68	267	268	17.6	16.5	17	16.1	231	143	3.9	1.7	13.8
NMNZM.118421	O'Shea [16]	New Zealand	135.5	97	62.5	23.9	12.3	142	532	418	17.6	16.5	21.3	21.3	217	150	4.1	2.2	14.6
NMNZM.118305	O'Shea [16]	New Zealand	121.5	55.5	52	23.9	12.3	107	393	360	17.6	16.5	22.9	17	217	162	3	1.2	14.6
NMNZM.118425	O'Shea [16]	New Zealand	89.5	59.5	37.2	23.9	12.3	62	365	192	17.6	16.5	11.5	11.6	217	142	3	1.5	14.6
310 6-83-1	AM	Western Australia	90	74.6	56.1	27.2	12.9	110	557	469	17.3	15.8	16	14.9	257	201	4.5	1.6	24.3
F200330	MV	Western Australia	95.8	79.1	53.9	23.7	11.2	114	420	444	16.7	15.9	15.1	14.3	281	209	3.5	1.7	14.3
F160306	MV	Western Australia	91.7	76.1	54.5	26.3	14.3	115	458	421	17.3	15.8	15.4	14.9	250	177	4.2	1.9	18.7
F200327	MV	Western Australia	111.7	66.6	52.2	22.9	12.6	98	458	426	17.3	15.8	15.4	14.9	283	192	4.4	1.6	13.5
F200329	MV	Western Australia	114.2	74.6	56.4	30.3	13.1	96	381	384	17.9	15.6	15	15.5	230	177	4.6	2.4	14.7
F200328	MV	Western Australia	163.4	83.5	65.8	34.3	14.1	107	559	544	17.3	15.8	15.4	14.9	291	218	3.9	1.7	26.8
F200326a	MV	Western Australia	127	68	54	25.7	12.4	127	365	520	17.6	16.5	17	16.1	217	207	5	2.1	11.9

Missing data (shown in bold) has been replaced by the global mean of the respective trait.

Institutions – AM  =  Australian Museum, Sydney, MV  =  Museum Victoria.

DFA showed a significant difference among individuals from east Australia, Tasmania, New Zealand and Western Australia (Pillai Trace =  1.201, F = 16.020, *df* = 6, 64, p = <0.001). DFA assigned 100% (n = 7) of male individuals from Western Australia to a single group comprised solely of Western Australian individuals (Table 3). DFA assigned 83% (n = 15) of east Australian individuals to the east Australian group, with 17% (n = 3) allocated to the Tasmanian group. Furthermore, 88% (n = 7) of Tasmanian individuals were assigned to the Tasmanian group, whilst 12% (n = 1) were grouped with east Australian individuals. All individuals from New Zealand (n = 3) were allocated into the east Australian group.

**Table 3:** Male Discriminant Function Analysis: Jackknifed classification matrix.

	East Australia	Tasmania	New Zealand	Western Australia	% correct
East Australia	15	0	3	0	83
Tasmania	3	0	0	0	0
New Zealand	1	0	7	0	88
Western Australia	0	0	0	7	100

Ranking of CLs determined male SCR3 to be the most important variable in detecting variance among groups (Table S8 in File S1). Step-down analysis performed on male SCR3 showed a significant difference among coasts (F = 41.775, *df* = 3, p = <0.001). Tukey's *post-hoc* analysis showed no significant difference among east Australia, Tasmania and New Zealand (p = >0.6), however Western Australia differed significantly from all three of these locations (p = <0.001). Analysis of ALR3 (second highest ranked variable) showed a significant difference among coast once the co-variate and SCR3 were included in the model (F = 5.333, *df* = 3, p = 0.01). Tukey's *post-hoc* analysis showed no significant difference between individuals from east Australia, Tasmania and Western Australia (p = >0.1), whilst individuals from New Zealand differed significantly from both eastern and Western Australia (p = 0.02 and 0.01 respectively). Analysis of SCL3 (third highest ranked trait) showed no significant difference among coasts once the co-variate, SCR3 and ALR3 were included in the model (F = 0.410, *df* = 3, p = 0.7). Due to a non-significant result, stepdown analysis was discontinued.

#### Females

**Figure 5:**
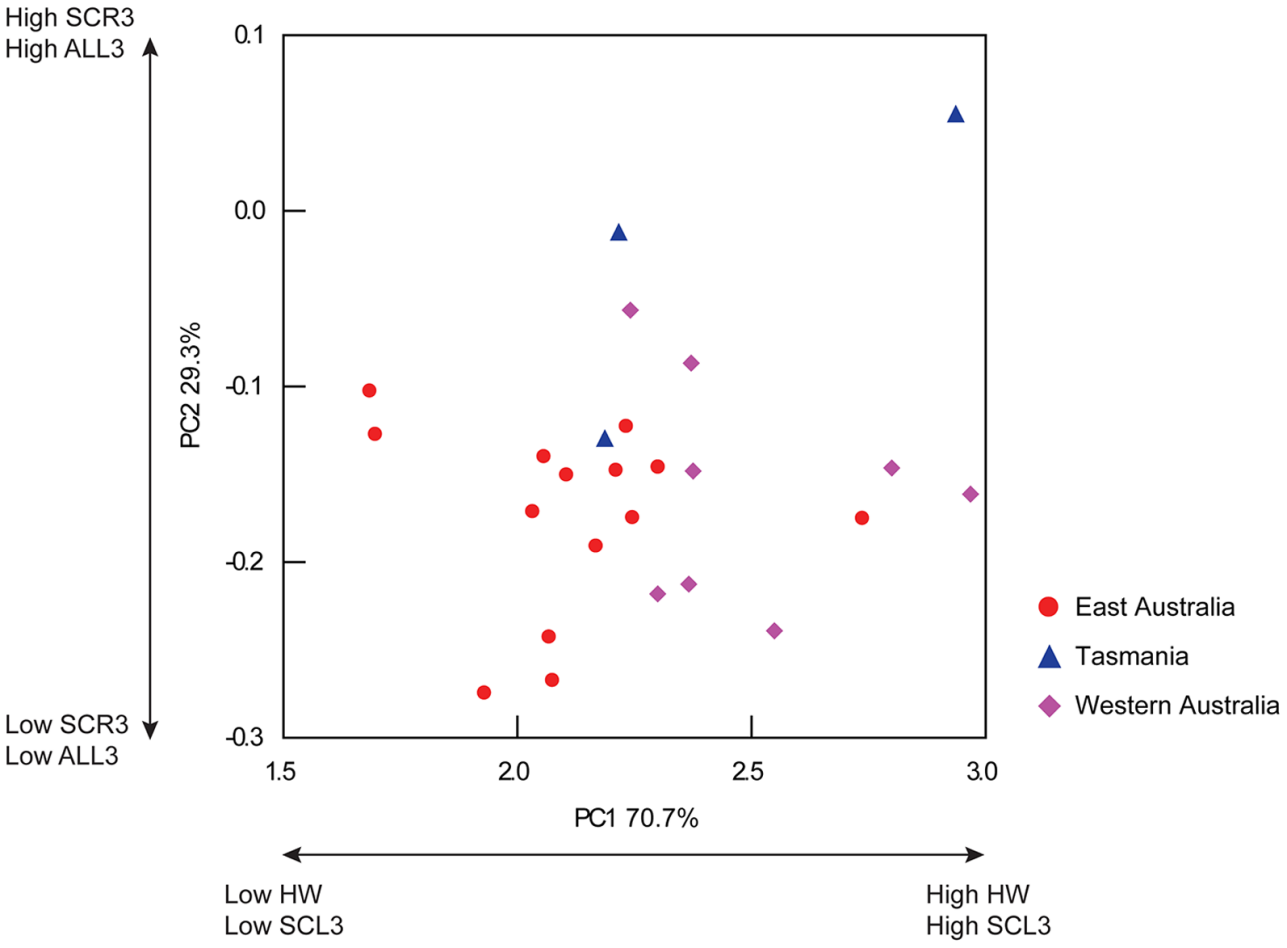
Principal component biplot of female individuals of the tetricus complex. X axis represents PC1 (explaining 70.7% of total variation) and is driven primarily by HW and SCL3. Y axis represents PC2 (explaining 29.3% of total variation) and is driven primarily by SCR3 and ALL3.

No interaction between the independent variable (coast) and the co-variate (MLd) was recorded (Pillai Trace  = 1.083, F = 1.574, *df* = 18, 24, p = >0.1), therefore the model was run without the interaction. No significant difference was recorded among three locations for the multivariate model based upon nine morphological traits (and MLd as co-variate) measured from 25 mature female octopods (Pillai Trace  = 0.122, F = 1.989, *df* = 18, 28, p = 0.05; Table 4). Visualisation of the female PC biplot showed overlap of individuals from east Australia, Tasmania and Western Australia along PC1 and PC2, which were primarily driven by HW/SCL3 and SCR3/ALL3 respectively (Figure 5). Although non-significant, female individuals from Western Australia generally possessed greater HW and SCL3 in relation to individuals from east Australia.

**Table 4:** Summary of mature female morphological measurements taken from preserved museum specimens of *Octopus tetricus* (east Australia and Tasmania), *O.* cf. *tetricus* (Western Australia) and *O. gibbsi* (New Zealand).

Catalogue #	Institution	Location	MLd	MW	HW	AW	SDn	WD	ALL3	ALR3	SCL3	SCR3
C156208	AM	East Australia	**67.6**	**65.4**	**44.6**	**20.7**	**12.2**	**84**	212	232	197	194
C171669	AM	East Australia	**62.1**	**65.4**	**44.6**	**20.7**	**12.2**	**84**	240	208	218	199
F78082	AM	East Australia	117.8	65.4	31.6	19.5	12	96	315	335	218	229
F78281a	AM	East Australia	70.3	**65.4**	**44.6**	**20.7**	**12.2**	**84**	323	209	235	222
F78283b	AM	East Australia	53.2	**65.4**	**44.6**	**20.7**	**12.2**	**84**	158	327	163	222
F80442a	MV	East Australia	96	64.9	34.7	16.8	9.5	69	358	356	247	234
F80442b	MV	East Australia	126.3	74.8	38.1	16.9	11.8	88	452	418	238	254
F80446	MV	East Australia	60.3	45.2	34.4	14.1	8.5	59	230	216	201	183
F85370a	MV	East Australia	100	**65.4**	**44.6**	**20.7**	**12.2**	**84**	287	400	216	221
F200320	MV	East Australia	120.2	**65.4**	**44.6**	**20.7**	**12.2**	**84**	323	435	235	224
F200319	MV	East Australia	104.5	80.1	52.2	28.8	15.2	100	437	409	265	290
F200318a	MV	East Australia	104.8	70.1	43.1	21	14.1	86	311	356	214	222
F200318c	MV	East Australia	84.4	47.7	31.5	13.4	9	52	259	243	204	198
F78283c	AM	East Australia	52.6	**65.4**	**44.6**	**20.7**	**12.2**	**84**	152	327	235	222
F180704	MV	Tasmania	102.2	64.9	42.8	20	12.5	74	323	369	235	230
F180701	MV	Tasmania	99.7	65.8	43.8	19.5	12.1	72	418	367	216	196
F180705	MV	Tasmania	135.4	88.7	57.5	25.7	17.2	99	524	327	302	222
NMNZM.90320	O'Shea [16]	New Zealand	48	35.7	30	**20.7**	6	45	141	313	206	194
NMNZM.131569	O'Shea [16]	New Zealand	48.9	28	27	**20.7**	5.9	40	283	141	235	145
NMNZM.118426	O'Shea [16]	New Zealand	137	90.5	55	**20.7**	16.8	112	424	485	235	243
F200334	MV	Western Australia	114.1	97	61.5	31.4	15	139	474	327	235	222
F160320	MV	Western Australia	84.2	59.2	44.6	20	20	75	304	327	264	264
F160321	MV	Western Australia	90.9	64.6	53.9	19.5	11.2	101	396	438	264	264
F160325	MV	Western Australia	97.3	59.3	48	17.5	9.7	82	350	355	257	266
F80447	MV	Western Australia	87.4	69.3	45.2	19.6	11.5	79	356	322	241	186
F200327	MV	Western Australia	88.3	64.3	51.9	20.4	12	82	240	277	290	253
F200331	MV	Western Australia	119.6	69	57.4	25.7	13.2	119	457	327	276	222
F160302	MV	Western Australia	71.4	68.9	51.8	22	13.2	97	302	305	236	200

Missing data (shown in bold) has been replaced by the global mean of the respective trait.

Institutions – AM  =  Australian Museum, Sydney, MV  =  Museum Victoria.

DFA showed a significant difference among individuals from east Australia, Tasmania and Western Australia (Pillai Trace =  0.678, F = 5.637, *df* = 4, 44, p = <0.01). DFA assigned 93% (n = 13) of east Australian female individuals into the correct group, whilst 7% (n = 1) were placed into the Western Australian group (Table 5). 67% of individuals from Tasmania (n = 2) were placed into the correct group, whilst 33% (n = 1) were considered to belong to the east Australian group. 38% (n = 3) of female individuals from Western Australia were correctly assigned, whilst 50% (n = 4) and 12% (n = 1) were assigned to east Australian and Tasmanian groups respectively.

**Table 5:** Female Discriminant Function Analysis: Jackknifed classification matrix.

	East Australia	Tasmania	Western Australia	% correct
East Australia	13	0	1	93
Tasmania	1	2	0	67
Western Australia	4	1	3	38

## Discussion

### Species level relationships

The main focus of this study was to resolve the taxonomic status of the Australasian tetricus complex. Molecular and morphological results are consistent with the hypothesis that disjunct populations of *Octopus tetricus* from Australia's east coast (including Tasmania), and from Western Australia are separate species. In addition, findings of this study support the hypothesis that *O. gibbsi* of New Zealand is synonymous with east Australian *O. tetricus* [2]. Consequently, we propose that the species name *O. gibbsi* be considered a junior synonym of *O. tetricus* Gould, 1852, and will hereafter be included in reference to *O. tetricus*.

In the present study, interspecific variation of *COI* between eastern *Octopus tetricus* and western *O.* cf. *tetricus* was over one order of magnitude (∼18 times) greater than intraspecific variation within each of these populations; a marked ‘barcoding gap’ consistent with the ‘ten times rule’ of Hebert *et al*., [48]. This study estimated interspecific divergence of *COI* sequences between *O. tetricus* and *O.* cf. *tetricus* to be 3.4%, similar to congeneric differences previously reported for octopods [49], [50]. For example, within the family Octopodidae interspecific variation was found to be 1–2% and 2–3.3% for the octopod genera *Pareledone* [51] and *Thaumeledone* [49] respectively. The interspecific variation found between *O. tetricus* and *O.* cf. *tetricus* (3.4%) displayed higher species-level differentiation than the 1.3% divergence recommended by Undheim *et al*., [50] for *O. vulgaris*. Low nucleotide sequence divergence between octopod species in this and previous studies contrasts with higher levels recorded among moths, butterflies and birds, which range from 5.8–9.1% [48], [52], [53].

GMYC analysis suggested Western Australian *Octopus* cf. *tetricus* is a distinct species from *O. tetricus*, as well as supporting the synonymy of *O. gibbsi* with *O. tetricus*. However, GMYC analysis detected a second cryptic Western Australian species, which conflicts with the phylogenetic and morphological results of this study (which show no such cryptic speciation). This may be due to gaps in knowledge (i.e. more species exist than is currently known), although more likely reflects the tendency for GMYC analyses to ‘over-split’ taxa [35].

Talavera *et al*., [35] investigated the ability of GMYC analysis to delineate species using the well resolved European butterflies. Their analysis revealed 16 unexpected cryptic species, which (although the authors acknowledged that at least some of these cryptic species may represent real entities) was considered to be a failure of the model due to the high levels of intraspecific variability recorded within butterflies. As interspecific variability between *Octopus* cf. *tetricus* and *O. tetricus* was far greater relative to the low intraspecific variability within each individual group (see above), the discovery of a second cryptic Western Australian species is considered likely to be an artefact of ‘over-splitting’ by GMYC analysis.

Multivariate morphological analyses showed congruence in detecting significant differences between individuals from east Australia/New Zealand and Western Australia; although females appear to be a less reliable morphological discriminator of species identity. Male morphology was able to successfully discriminate between *Octopus tetricus* and *O.* cf. *tetricus*. Sucker numbers on the males third right arm explained the most variation between *O. tetricus* and *O.* cf. *tetricus*, with *O.* cf. *tetricus* having significantly greater sucker numbers. Males third right arms (left in some species) possess the hectocotylus, a copulatory organ used to pass sperm to the female during mating. The hectocotylus is comprised of the ligula and calamus, which provide a limit to the emergence of new suckers at a relatively early stage of ontogeny [54]. Toll [54] investigated sucker counts on the males hectocotylised arm (HASC) among 12 species of the sub-family Octopodinae, and demonstrated its value in identification and delimitation of otherwise morphologically similar octopods. Toll [54] showed sucker numbers on the hectocotylised arm to be relatively fixed, with different species appearing to be characterised by a narrow range of values for HASC, which he proposed were genetically defined. This assumption appears to be supported by congruence between molecular and HASC data obtained in this study. Consistency of sucker counts despite fixation, preservation [54] or environmental influence further reinforces the usefulness of male HASC in cryptic cephalopod taxonomy.

### Biogeographic factors

Speciation between *Octopus tetricus* and *O.* cf. *tetricus* is likely the result of reproductive isolation due to allopatric eastern and western distributions. Divergence of *O. tetricus* (east Australian, Tasmania and New Zealand populations) and *O.* cf. *tetricus* (from Western Australia) were estimated to have occurred somewhere within the last 3.2–6.9 million years. This coincides with cooling of the previously tropical Miocene seas along the southern Australian coastline and the rising of the Bassian Isthmus (a historic land-bridge joining Tasmania and mainland Australia) during the Pliocene era, potentially dividing populations of a common tetricus complex ancestor in two. Glacial-interglacial epochs during the early Pleistocene resulted in northward progression of cooler waters, initiating the retreat of numerous wide-spread subtropical species along the eastern and western coasts, isolating populations which allowed for genetic differentiation to commence [55].

More recently oceanographic, climatic and ecological factors have likely maintained contemporary disjunction following the final inundation of the Bassian Isthmus 14,000 years ago. For example, the southern coast of Australia possesses extensive expanses with limited reef habitat in the Great Australian Bight and east of Wilson's Promontory in south-east Victoria. Limited reef habitat has been proposed as a factor in genetic divergence of populations and speciation events in other southern marine taxa such as decapods, echinoderms [56], [57], and gastropods [58], [59]. However studies conducted on *O. gibbsi* (treated as *O. tetricus*) among reefs in Northern New Zealand found reef habitat was not essential for successful settlement [17], and *O. tetricus* were often found in lairs within sandy bottomed estuaries along the southern coast of New South Wales (M. Amor, personal observation). The Great Australian Bight is also associated with sharp drops in sea surface temperature (SST), which is a likely explanation for maintenance of allopatric distributions between east and west taxa.

The absence of significant genetic differentiation between New Zealand and east Australian *Octopus tetricus* populations suggests ongoing gene flow across the Tasman Sea; a 2000 km wide marine body separating the two landmasses. Due to the benthic shallow-water habit of *Octopus tetricus* adults [15], connectivity between New Zealand and east Australian populations is likely attributable to trans-Tasman dispersal during the planktonic larval stage; although adults of the genus *Octopus* can raft on floating wood or drifting macroalgae [60], which may function as a rare mode of passive trans-Tasman migration.

A number of other southern Australasian marine taxa display similar trans-Tasman genetic homogeneity, including the southern rock lobster, *Jasus edwardsii* [61], [62], [63] and morwong (cheilodactylid) fishes [64], [65]. Planktonic larval durations (PLD) for the Octopodinae appear much shorter (35–60 days; reviewed in Villanueva, [66]) than those of the lobster *J. edwardsii* (2 years [67]) and cheilodactylid fishes (1 year [68]). *Octopus* paralarvae appear to be active and often constant swimmers [13], [69], potentially facilitating dispersal within surface currents. However, simulation based oceanographic modelling studies suggests that in the absence of rafting, a period of several months is required for even a low probability of successful trans-Tasman dispersal [70]. Octopod paralarvae have been observed rafting on macroalgal and other drift debris [71], which may function as habitat for post-settlement juveniles until arrival at suitable shallow-water habitat. Additionally, paralarvae of some octopods can delay settlement in the absence of suitable habitat [72]. These ‘super-paralarvae’ obtain larger sizes and more developed swimming capabilities, while retaining paralarval morphological characters (reviewed in Villanueva and Norman, [69]), and may facilitate trans-Tasman dispersal for *Octopus tetricus*. Further investigation into physiological, behavioural and ecological aspects of paralarval life histories would further our understanding of the dispersive capabilities of *O. tetricus*.

### Evidence of range shifts and implications of climate change

This study is the first to verify the presence of *Octopus tetricus* in the temperate waters off Flinders Island, Tasmania. This suggests the southern distributional limit of *O. tetricus* along the Australian mainland (currently recognised as Eden, New South Wales) is underestimated and requires resurveying, in fact *O. tetricus* has been sighted as far south as Cape Conran, Victoria (M. Amor personal observation, 2013). Temperate coastal waters in eastern Tasmania appear to be warming at approximately four times the global ocean warming average due to climate change driven strengthening of the Eastern Australian Current [73]. This has been linked to recent range expansions of a number of sub-tropical and tropical marine species in Tasmanian waters, including 22 fish species, eastern rock lobster, leatherback turtle and two species of box jellyfish [74]. Coastal warming in Tasmania may have resulted in current temperatures exceeding the lower thermal limits of *O. tetricus* paralarvae, potentially allowing population establishment outside of their previously known range, as has been suggested for the sea urchin *Centrostephanus rodgersii* [75]. Investigation of the potential impacts of *O.tetricus* range expansion on native ecosystems and commercial fisheries should be given high priority.

### Broader phylogenetic relationships

Mitochondrial DNA analyses placed the Australasian tetricus complex within a monophyletic clade along with Japanese and Chinese *Octopus vulgaris*, supporting previous speculations that these taxa are closely related [76]. The current study estimated that the tetricus complex and Japanese/Chinese *O. vulgaris* arose from a common ancestor following an ‘anti-tropical’ divergence event that took place between ∼5.4–11.7 ma. This estimated time of divergence is consistent with mid-Miocene climatic warming and the emergence of intervening tropical waters at lower latitudes [77]; suggesting vicariant isolation of a once common subtropical ancestor into Northern and Southern Hemisphere populations. Warming of equatorial waters during the mid-Miocene has also been implicated in trans-equatorial divergences for a number of marine taxa, especially reef fishes [78], [79], [80]. In addition, anti-tropical affinities between other subtropical Australasian-Japanese/Asian octopods have been noted. For example, *Amphioctopus kagoshimensis* Ortmann, 1888 from subtropical Japan and the morphologically indistinguishable taxon *Amphioctopus* cf. *kagoshimensis* recently discovered at similar latitudes in Australasian waters are predicted to represent closely related relicts of a wider distributed ancestry [76]. The ability of molecular analyses to detect cryptic species suggests that future molecular work would clarify the taxonomic, phylogenetic and palaeogeographical relationships between seemingly cryptic anti-tropical cephalopod species pairs.

Paraphyletic relationships within the vulgaris complex revealed in this study directly question the purported cosmopolitan distribution of *Octopus vulgaris,* and supports hypotheses regarding the existence of numerous cryptic vulgaris-like species [2], [76], [81]. Norman and Kubodera [76] previously suggested the possibility of an Asian vulgaris-like species ranging from Taiwan to Japan that was distinctly separate from genuine *O. vulgaris*, originally described from the Mediterranean Sea and Atlantic Ocean. Findings of this study support this theory of speciation between Atlantic and Pacific vulgaris-like species. However, the results of this study were based on samples from extremes in the distribution of *O. vulgaris*. Future work aimed at resolving the taxonomy of this species complex should include individuals from a representative range of the entire *O. vulgaris* distribution.

### Conclusions and future directions

This study is the first attempt to resolve the taxonomy of the Australasian *Octopus tetricus* species complex. Molecular and morphological results support east Australian *Octopus tetricus* as a distinct species from Western Australian *O.* cf. *tetricus*, which requires future formal taxonomic description. Additionally, New Zealand's *O. gibbsi* was found to be synonymous with east Australian and Tasmanian *O. tetricus.* Paraphyletic relationships within the *Octopus vulgaris* complex revealed in this study adds support to hypotheses regarding the existence of numerous cryptic vulgaris-like species, warranting taxonomic revision of the *O. vulgaris* species complex to aid in the management of this significant global marine resource.

## Supporting Information

File S1 **Supporting tables.** Table S1, Specimen information for individuals of which molecular sequencing was undertaken during the present study. Table S2, Specimen information for individuals accessed via GenBank for use in the present study. Table S3, Specimen information for individuals of which morphological traits were recorded during the present study. Table S4, Canonical correlation (CC) output for male octopod multivariate analysis. Table S5, Canonical loadings (CL) output for male octopod multivariate analysis. Table S6, Principal components (PC) calculated from canonical correlation and canonical loading outputs from male octopod multivariate analysis. Table S7, Eigenvalues and principal component (PC) variance contribution outputs from male octopod multivariate analysis. Table S8, Ranked canonical loadings (CL) from male octopod multivariate analysis; based upon contribution to principal components (PC). Table S9, Canonical correlation (CC) output for female octopod multivariate analysis. Table S10, Canonical loadings (CL) output for female octopod multivariate analysis. Table S11, Principal components (PC) calculated from canonical correlation and canonical loading outputs from female octopod multivariate analysis. Table S12, Eigenvalues and principal component (PC) variance contribution outputs from female octopod multivariate analysis. Table S13, Timing of divergence estimates (Tamura-Nei genetic distance) for *Octopus tetricus* (East Australia and New Zealand) and *O.* cf. *tetricus* (Western Australia). Table S14, Timing of divergence estimates (Tamura-Nei genetic distance) for the Australasian tetricus complex and Japanese/Chinese representatives of the *Octopus vulgaris* group.
(DOCX)
