# Supplementary material for: Allopatric Speciation within a Cryptic Species Complex of Australasian Octopuses
Source: PLoS One. 2014 Jun 25;9(6):e98982. doi: 10.1371/journal.pone.0098982 (PMC4070898; doi:10.1371/journal.pone.0098982)
Supplement: File S1 — Supporting tables. Table S1, Specimen information for individuals of which molecular sequencing was undertaken during the present study. Table S2, Specimen information for individuals accessed via GenBank for use in the present study. Table S3, Specimen information for individuals of which morphological traits were recorded during the present study. Table S4, Canonical correlation (CC) output for male octopod multivariate analysis. Table S5, Canonical loadings (CL) output for male octopod multivariate analysis. Table S6, Principal components (PC) calculated from canonical correlation and canonical loading outputs from male octopod multivariate analysis. Table S7, Eigenvalues and principal component (PC) variance contribution outputs from male octopod multivariate analysis. Table S8, Ranked canonical loadings (CL) from male octopod multivariate analysis; based upon contribution to principal components (PC). Table S9, Canonical correlation (CC) output for female octopod multivariate analysis. Table S10, Canonical loadings (CL) output for female octopod multivariate analysis. Table S11, Principal components (PC) calculated from canonical correlation and canonical loading outputs from female octopod multivariate analysis. Table S12, Eigenvalues and principal component (PC) variance contribution outputs from female octopod multivariate analysis. Table S13, Timing of divergence estimates (Tamura-Nei genetic distance) for Octopus tetricus (East Australia and New Zealand) and O. cf. tetricus (Western Australia). Table S14, Timing of divergence estimates (Tamura-Nei genetic distance) for the Australasian tetricus complex and Japanese/Chinese representatives of the Octopus vulgaris group. (DOCX) [file pone.0098982.s001.docx]

# Citation: Amor, M.D., M.D. Norman, H.E. Cameron and J.M. Strugnell. Allopatric speciation within a cryptic species complex of Australasian octopuses.

# Supplementary data

Table S1: Specimen information for individuals of which molecular sequencing was undertaken during the present study.

| **Sample** | **Species** | **Location** | **Region** | ***12S*** | ***16S*** | ***COI*** | ***COIII*** | ***Cytb*** |
| --- | --- | --- | --- | --- | --- | --- | --- | --- |
| NQ001 | *O.tetricus* | NSW | Wallaga Lake | * | * | * | * |  |
| NQ002 | *O.tetricus* | NSW | Wallaga Lake | * |  | * | * | * |
| NQ003 | *O.tetricus* | NSW | Wallaga Lake |  | * | * | * | * |
| NQ010 | *O.tetricus* | NSW | Wallaga Lake | * |  | * | * | * |
| NQ011 | *O.tetricus* | NSW | Wallaga Lake |  | * | * | * | * |
| NQ015 | *O.tetricus* | NSW | Narooma | * | * | * | * |  |
| NQ028 | *O.tetricus* | NSW | Shoal Bay, Port Stephens |  |  | * | * | * |
| NQ029 | *O.tetricus* | NSW | Shoal Bay, Port Stephens | * | * | * | * |  |
| TAS4113 | *O.tetricus* | Tasmania | Flinders Island |  |  | * | * |  |
| TAS4123 | *O.tetricus* | Tasmania | Flinders Island | * |  | * | * |  |
| TAS4124 | *O.tetricus* | Tasmania | Flinders Island |  |  | * | * |  |
| TAS4126 | *O.tetricus* | Tasmania | Flinders Island |  |  | * | * |  |
| TAS4132 | *O.tetricus* | Tasmania | Flinders Island |  |  |  | * |  |
| LML1 | *O. gibbsi* | NZ | Leigh Marine Lab | * | * | * | * | * |
| LML4 | *O. gibbsi* | NZ | Leigh Marine Lab | * | * | * | * | * |
| LML8 | *O. gibbsi* | NZ | Leigh Marine Lab | * | * | * | * | * |
| NZ1 | *O. gibbsi* | NZ | Leigh Marine Lab | * | * | * | * | * |
| ct123 | *O.*cf. *tetricus* | WA | Woodmans Point | * | * | * | * | * |
| ct133 | *O.*cf. *tetricus* | WA | Town Jetty, Albany | * | * | * | * | * |
| SWA006 | *O.*cf. *tetricus* | WA | Lucky Bay, Cape Le Grand |  |  | * | * | * |
| SWA007 | *O.*cf. *tetricus* | WA | Lucky Bay, Cape Le Grand |  | * | * | * | * |
| SWA008 | *O.*cf. *tetricus* | WA | Lucky Bay, Cape Le Grand |  |  | * | * | * |
| SWA009 | *O.*cf. *tetricus* | WA | Esperance |  | * | * | * | * |
| SWA010 | *O.*cf. *tetricus* | WA | Esperance |  | * | * | * | * |
| WAM6701 | *O.*cf. *tetricus* | WA | Mandurah |  |  | * | * |  |
| WAM6702 | *O.*cf. *tetricus* | WA | Mandurah |  |  | * | * |  |
| WAM6703 | *O.*cf. *tetricus* | WA | Mandurah |  |  | * | * |  |
| WAM6704 | *O.*cf. *tetricus* | WA | Mandurah |  | * | * | * | * |
| WAM6705 | *O.*cf. *tetricus* | WA | Mandurah |  |  | * |  |  |
| WAM6706 | *O.*cf. *tetricus* | WA | Mandurah |  | * | * | * | * |
| WAM6707 | *O.*cf. *tetricus* | WA | Mandurah |  | * | * | * | * |
| WAM6708 | *O.*cf. *tetricus* | WA | Mandurah |  |  | * | * |  |
| WAM6709 | *O.*cf. *tetricus* | WA | Mandurah |  |  | * | * |  |
| WAM6710 | *O.*cf. *tetricus* | WA | Mandurah |  | * | * | * | * |
| SAVULG01 | *O.vulgaris* | South Africa | Port Elizabeth |  |  | * |  |  |
| OVAL1 | *O.vulgaris* | Spain | Perpignan |  |  | * | * | * |
| OVAL2 | *O.vulgaris* | Spain | Perpignan |  |  | * | * | * |
| OVAL3 | *O.vulgaris* | Spain | Perpignan |  |  | * | * | * |
| OVAL4 | *O.vulgaris* | Spain | Perpignan |  |  | * | * | * |
| OVAL5 | *O.vulgaris* | Spain | Perpignan |  |  | * | * | * |

*Locations –* NSW = New South Wales, NZ = New Zealand, WA = Western Australia

Table S2: Specimen information for individuals accessed via GenBank for use in the present study.

| **Accession #** | **Species** | **Location** | **Gene(s)** |
| --- | --- | --- | --- |
| AJ390318 | *O. mimus* | Chile | *16S* |
| AJ012128 | *O. mimus* | Chile | *COIII* |
| AJ250480 | *O. mimus* | Costa Rica | *16S* |
| AJ390319 | *O. mimus* | Costa Rica | *COIII* |
| HQ846021 | *O. vulgaris* | China | *16S* |
| HQ846110 | *O. vulgaris* | China | *COI* |
| HQ846061 | *O. vulgaris* | China | *16S* |
| HQ846154 | *O. vulgaris* | China | *COI* |
| NC006353 | *O. vulgaris* | Japan | *12S, 16S, COI, COIII, Cytb* |
| AB430546 | *O. vulgaris* | Japan | *COI* |
| AB573217 | *O. vulgaris* | Japan | *COIII* |
| AB430547 | *O. vulgaris* | Japan | *COI* |
| AB573219 | *O. vulgaris* | Japan | *COIII* |
| AB430548 | *O. vulgaris* | Japan | *COI* |
| AB573218 | *O. vulgaris* | Japan | *COIII* |
| FN424379 | *O. vulgaris* | St Paul and Amsterdam Islands | *COI* |
| FN424382 | *O. vulgaris* | St Paul and Amsterdam Islands | *COIII* |
| FN424380 | *O. vulgaris* | St Paul and Amsterdam Islands | *COI* |
| FN424383 | *O. vulgaris* | St Paul and Amsterdam Islands | *COIII* |
| FN424381 | *O. vulgaris* | St Paul and Amsterdam Islands | *COI* |
| FN424384 | *O. vulgaris* | St Paul and Amsterdam Islands | *COIII* |
| AJ628241 | *O. vulgaris* | South Africa | *COIII* |
| AJ628204 | *O. vulgaris* | South Africa | *Cytb* |
| DQ683234 | *O. vulgaris* | South Africa, Durban | *16S* |
| DQ683214 | *O. vulgaris* | South Africa, Durban | *COI* |
| DQ683235 | *O. vulgaris* | South Africa, Durban | *16S* |
| DQ683215 | *O. vulgaris* | South Africa, Durban | *COI* |
| DQ683236 | *O. vulgaris* | South Africa, Durban | *16S* |
| DQ683216 | *O. vulgaris* | South Africa, Durban | *COI* |
| DQ683237 | *O. vulgaris* | South Africa, Durban | *16S* |
| DQ683217 | *O. vulgaris* | South Africa, Durban | *COI* |
| DQ683238 | *O. vulgaris* | South Africa, Durban | *16S* |
| DQ683218 | *O. vulgaris* | South Africa, Durban | *COI* |
| DQ683239 | *O. vulgaris* | South Africa, Durban | *16S* |
| DQ683219 | *O. vulgaris* | South Africa, Durban | *COI* |
| DQ683247 | *O. vulgaris* | Spain, Galicia | *16S* |
| DQ683221 | *O. vulgaris* | Spain, Galicia | *COI* |
| DQ683230 | *O. vulgaris* | South Africa, Hout Bay | *16S* |
| DQ683208 | *O. vulgaris* | South Africa, Hout Bay | *COI* |
| DQ683228 | *O. vulgaris* | South Africa, Port Elizabeth | *16S* |
| DQ683212 | *O. vulgaris* | South Africa, Port Elizabeth | *COI* |
| DQ683232 | *O. vulgaris* | South Africa, Struisbaai | *16S* |
| DQ683210 | *O. vulgaris* | South Africa, Struisbaai | *COI* |
| DQ683244 | *O. vulgaris* | Africa, Senegal | *16S* |
| DQ683224 | *O. vulgaris* | Africa, Senegal | *COI* |
| DQ683241 | *O. vulgaris* | Tristan da Chuna | *16S* |
| DQ683205 | *O. vulgaris* | Tristan da Chuna | *COI* |
| DQ683240 | *O. vulgaris* | South Africa, Umhlanga | *16S* |
| DQ683220 | *O. vulgaris* | South Africa, Umhlanga | *COI* |

Table S3: Specimen information for individuals of which morphological traits were recorded during the present study.

| **Catalogue #** | **Institution** | **Species** | **Coast** | **Location** | **Latitude, longitude** |
| --- | --- | --- | --- | --- | --- |
| C126244 | AM | *O. tetricus* | East | Nelson Head, Port Stephens, NSW | -32.716667, 152.166667 |
| C156208 | AM | *O. tetricus* | East | Shelly Beach, North Manly, NSW | -33.800000, 151.300000 |
| C171669 | AM | *O. tetricus* | East | Parsley Bay, NSW | -35.866667, 151.283333 |
| C171685 | AM | *O. tetricus* | East | Woody Head, Iluka, NSW | -29.358015, 153.354721 |
| F78082 | AM | *O. tetricus* | East | Merewether, NSW | -32.98333, 151.7833300 |
| F78281a | AM | *O. tetricus* | East | Newcastle, NSW | -32.916667, 151.950000 |
| F78283b | AM | *O. tetricus* | East | Newcastle, NSW | -32.866667, 152.016667 |
| F160334 | MV | *O. tetricus* | East | Wallaga Lake, NSW | -36.350000, 150.050000 |
| F182057 | MV | *O. tetricus* | East | Narooma Inlet, NSW | -36.218238, 150.132300 |
| F182058 | MV | *O. tetricus* | East | Narooma Inlet, NSW | -36.218238, 150.132300 |
| F200317 | MV | *O. tetricus* | East | Wreck Bay, NSW | -34.200000, 150.716667 |
| F200318a | MV | *O. tetricus* | East | Wreck Bay, NSW | -35.216667, 150.716667 |
| F200318b | MV | *O. tetricus* | East | Wreck Bay, NSW | -35.216667, 150.716667 |
| F200318c | MV | *O. tetricus* | East | Wreck Bay, NSW | -35.216667, 150.716667 |
| F200319 | MV | *O. tetricus* | East | Long Reef, Sydney, NSW | -33.733333, 151.316667 |
| F200319 | MV | *O. tetricus* | East | Long Reef, Sydney, NSW | -33.733333, 151.316667 |
| F200320 | MV | *O. tetricus* | East | Merimbula Harbour, NSW | -36.883333, 149.916667 |
| F200321 | MV | *O. tetricus* | East | Shelly Beach, North Manly, NSW | -33.800000, 151.300000 |
| F200323 | MV | *O. tetricus* | East | Shelly Beach, North Manly, NSW | -33.800000, 151.300000 |
| F200324 | MV | *O. tetricus* | East | Long Reef, Sydney, NSW | -33.733333, 151.316667 |
| F77273 | MV | *O. tetricus* | East | Newcastle, NSW | -32.966667, 151.783333 |
| F77274 | MV | *O. tetricus* | East | Tathra, NSW | -36.616667, 150.066667 |
| F78281(B) | MV | *O. tetricus* | East | Newcastle, NSW | -32.933333, 151.950000 |
| F78283c | MV | *O. tetricus* | East | Newcastle, NSW | -32.866667, 152.016667 |
| F80438 | MV | *O. tetricus* | East | Amity Point, North Stradbroke Island, QLD | -27.398309, 153.442056 |
| F80439 | MV | *O. tetricus* | East | Ned’s Beach, Lord Howe Island, NSW | -31.524986, 159.060767 |
| F80440 | MV | *O. tetricus* | East | North Stradbroke Island, QLD | -27.668056, 153.484722 |
| F80442a | MV | *O. tetricus* | East | Sydney, NSW | -33.867487, 151.206990 |
| F80442b | MV | *O. tetricus* | East | Sydney, NSW | -33.867487, 151.206990 |
| F80445 | MV | *O. tetricus* | East | Potters Point, Karnell, NSW | -34.045000, 151.211667 |
| F80446 | MV | *O. tetricus* | East | Potters Point, Karnell, NSW | -34.040000, 151.211667 |
| F85370a | MV | *O. tetricus* | East | Wreck Bay, NSW | -35.200000, 150.733333 |
| F180696 | MV | *O. tetricus* | TAS | Flinders Island, TAS | -39.772767, 148.470439 |
| F180697 | MV | *O. tetricus* | TAS | Flinders Island, TAS | -39.772767, 148.470437 |
| F180698 | MV | *O. tetricus* | TAS | Flinders Island, TAS | -39.772767, 148.470440 |
| F180699 | MV | *O. tetricus* | TAS | Flinders Island, TAS | -39.772767, 148.470433 |
| F180700 | MV | *O. tetricus* | TAS | Flinders Island, TAS | -39.772767, 148.470435 |
| F180701 | MV | *O. tetricus* | TAS | Flinders Island, TAS | -39.772767, 148.470441 |
| F180702 | MV | *O. tetricus* | TAS | Flinders Island, TAS | -39.772767, 148.470444 |
| F180704 | MV | *O. tetricus* | TAS | Flinders Island, TAS | -39.772767, 148.470438 |
| F180705 | MV | *O. tetricus* | TAS | Flinders Island, TAS | -39.772767, 148.470443 |
| F180706 | MV | *O. tetricus* | TAS | Flinders Island, TAS | -39.772767, 148.470436 |
| F180707 | MV | *O. tetricus* | TAS | Flinders Island, TAS | -39.772767, 148.470442 |
| 310 6-83-1 | AM | *O.* cf. *tetricus* | West | Fathom Bank, off Garden Island, WA | -32.242955, 115.698630 |
| F160302 | MV | *O.* cf. *tetricus* | West | Busselton Jetty, WA | -33.650000, 155.333333 |
| F160306 | MV | *O.* cf. *tetricus* | West | Esperance boat wharf, WA | -33.000000, 121.000000 |
| F160320 | MV | *O.* cf. *tetricus* | West | Esperance tanker jetty, WA | -33.868611, 121.903889 |
| F160321 | MV | *O.* cf. *tetricus* | West | Esperance tanker jetty, WA | -33.868611, 121.903889 |
| F160325 | MV | *O.* cf. *tetricus* | West | Princess royal harbour, Albany, WA | -35.075000, 117.925000 |
| F200326a | MV | *O.* cf. *tetricus* | West | Busselton jetty, WA | -33.650000, 155.333333 |
| F200327 | MV | *O.* cf. *tetricus* | West | Lucky bay, Cape Le Grand National Park, WA | -33.970413, 122.269592 |
| F200327 | MV | *O.* cf. *tetricus* | West | Lucky bay, Cape Le Grand National Park, WA | -33.970413, 122.269592 |
| F200328 | MV | *O.* cf. *tetricus* | West | Peaceful bay, 30 km East of Walpole WA | -35.041944, 116.930752 |
| F200329 | MV | *O.* cf. *tetricus* | West | Rockingham Grain Jetty, WA | -32.255945, 115.751492 |
| F200330 | MV | *O.* cf. *tetricus* | West | Rockingham Grain Jetty, WA | -32.255945, 115.751492 |
| F200331 | MV | *O.* cf. *tetricus* | West | Esperance tanker jetty, WA | -33.868611, 121.903889 |
| F200334 | MV | *O.* cf. *tetricus* | West | Town jetty, Albany, WA | -35.030625, 117.886519 |
| F80447 | MV | *O.* cf. *tetricus* | West | Woodmans Point, Perth, WA | -32.125398, 115.758562 |
|  |  |  |  |  |  |

*Institutions – AM = Australian Museum, Sydney, MV = Museum Victoria*

*Locations –* NSW = New South Wales, QLD = Queensland TAS = Tasmania, WA = Western Australia

Table S4: Canonical correlation (CC) output for male octopod multivariate analysis.

| **CC** | **Value** |
| --- | --- |
| 1 | 0.943 |
| 2 | 0.774 |
| 3 | 0.762 |

Table S5: Canonical loadings (CL) output for male octopod multivariate analysis.

| **Trait** | **CL1** | **CL2** | **CL3** |
| --- | --- | --- | --- |
| MW | 0.073 | 0.07 | -0.07 |
| HW | 0.196 | 0.259 | -0.131 |
| AW | 0.14 | 0.238 | -0.371 |
| SDn | 0.05 | 0.121 | -0.304 |
| WD | 0.181 | 0.353 | -0.152 |
| ALL3 | 0.058 | -0.217 | 0.425 |
| ALR3 | 0.468 | -0.274 | -0.16 |
| LSDL2 | -0.055 | -0.014 | -0.236 |
| LSDL3 | -0.119 | -0.097 | -0.305 |
| LSDR2 | -0.158 | 0.025 | -0.302 |
| LSDR3 | -0.139 | -0.014 | -0.382 |
| SCL3 | 0.256 | -0.103 | 0.312 |
| SCR3 | 0.686 | -0.033 | 0.435 |
| LL | 0.068 | -0.287 | -0.252 |
| CL | -0.017 | -0.233 | -0.09 |
| TOL | 0.096 | -0.165 | 0.017 |

Table S6: Principal components (PC) calculated from canonical correlation and canonical loading outputs from male octopod multivariate analysis.

| **Catalogue number** | **Location** | **PC1** | **PC2** | **PC3** |
| --- | --- | --- | --- | --- |
| C126244 | East Australia | 1.398564154 | -0.063633217 | -1.82498345 |
| C171685 | East Australia | 1.656582137 | -0.099131475 | -1.168465778 |
| F160334 | East Australia | 1.574527919 | 0.363012939 | -1.085086723 |
| F77273 | East Australia | 1.685109747 | -0.300965674 | -0.96476396 |
| F77274 | East Australia | 1.568450827 | -0.501585849 | -1.131766839 |
| F78281(B) | East Australia | 1.351409537 | 0.050502997 | -1.693015773 |
| F80438 | East Australia | 1.710610872 | -0.313058649 | -1.481486248 |
| F80439 | East Australia | 1.622998216 | -0.385155637 | -0.850786645 |
| F80440 | East Australia | 1.818444188 | -0.611405995 | -1.468833801 |
| F80445 | East Australia | 1.467906926 | -0.369658284 | -1.688319729 |
| F200319 | East Australia | 1.790746655 | -0.313842653 | -1.63515882 |
| F200324 | East Australia | 1.592004277 | -0.221444375 | -1.720480192 |
| F200323 | East Australia | 1.522560376 | -0.168410585 | -1.272437234 |
| F200321 | East Australia | 1.362814495 | -0.009377961 | -1.689063827 |
| F182058 | East Australia | 1.912142313 | -0.183517245 | -2.880150654 |
| F182057 | East Australia | 1.916011784 | -0.192024188 | -2.626587877 |
| F200317 | East Australia | 1.670192661 | -0.389328693 | -3.064599549 |
| F200318b | East Australia | 1.679143563 | -0.506436436 | -1.140385436 |
| F180706 | Tasmania | 1.710481088 | -0.548840695 | -1.922252402 |
| F180698 | Tasmania | 1.573629475 | -0.601183599 | -1.217745373 |
| F180707 | Tasmania | 1.893463296 | -0.91658773 | -2.227068944 |
| F180699 | Tasmania | 1.547096979 | -0.47192172 | -1.45772354 |
| F180700 | Tasmania | 1.661304028 | -0.506232288 | -1.473664311 |
| F180697 | Tasmania | 1.656125045 | -0.68637114 | -1.317081058 |
| F180696 | Tasmania | 1.396853251 | -0.53629949 | -1.298398701 |
| F180702 | Tasmania | 1.476770081 | -0.546380117 | -1.386584132 |
| NMNZM.118421 | New Zealand | 2.025951471 | -0.327920877 | -1.827612087 |
| NMNZM.118305 | New Zealand | 1.830826997 | -0.206752673 | -1.595982188 |
| NMNZM.118425 | New Zealand | 1.505001738 | -0.350295695 | -1.179311946 |
| 310 6-83-1 | Western Australia | 2.472296823 | -0.611333741 | -1.313158431 |
| F200330 | Western Australia | 2.385776448 | -0.391990469 | -1.225903028 |
| F160306 | Western Australia | 2.238991724 | -0.444423706 | -1.501335696 |
| F200327 | Western Australia | 2.244534044 | -0.520498612 | -1.278073611 |
| F200329 | Western Australia | 2.164884583 | -0.479896537 | -1.694166644 |
| F200328 | Western Australia | 2.795060035 | -0.557242263 | -1.37264284 |
| F200326a | Western Australia | 2.39664783 | -0.466762457 | -1.718671345 |

Table S7: Eigenvalues and principal component (PC) variance contribution outputs from male octopod multivariate analysis.

|  | **Eigenvalue** | **PC variance contribution (%)** |
| --- | --- | --- |
| 1 | 8.029263844 | 73.6 |
| 2 | 1.49423831 | 13.7 |
| 3 | 1.384608781 | 12.7 |
| Total | 10.90811093 | 100 |

Table S8: Ranked canonical loadings (CL) from male octopod multivariate analysis; based upon contribution to principal components (PC).

| **Trait** | **PC contribution** | **Rank** |
| --- | --- | --- |
| SCR3 | 50.95 | 1 |
| ALR3 | 38.20 | 2 |
| SCL3 | 20.25 | 3 |
| WD | 18.16 | 4 |
| HW | 17.98 | 5 |
| AW | 13.57 | 6 |
| SDeR2 | 11.97 | 7 |
| SDeR3 | 10.42 | 8 |
| SDeL3 | 10.09 | 9 |
| TOL | 9.33 | 10 |
| LL | 8.94 | 11 |
| ALL3 | 7.24 | 12 |
| MW | 6.33 | 13 |
| SDn | 5.34 | 14 |
| CL | 4.44 | 15 |
| SDeL2 | 4.24 | 16 |

Table S9: Canonical correlation (CC) output for female octopod multivariate analysis.

| **CC** | **Value** |
| --- | --- |
| 1 | 0.817 |
| 2 | 0.674 |

Table S10: Canonical loadings (CL) output for female octopod multivariate analysis.

| **Trait** | **CL1** | **CL2** |
| --- | --- | --- |
| MW | 0.098 | 0.067 |
| HW | 0.553 | 0.086 |
| AW | 0.176 | -0.006 |
| SDn | 0.177 | 0.158 |
| WD | 0.297 | -0.33 |
| ALL3 | 0.31 | 0.225 |
| ALR3 | 0.018 | -0.071 |
| SCL3 | 0.493 | 0.037 |
| SCR3 | 0.118 | -0.315 |

Table S11: Principal components (PC) calculated from canonical correlation and canonical loading outputs from female octopod multivariate analysis.

| **Catalogue number** | **Location** | **PC1** | **PC2** |
| --- | --- | --- | --- |
| C156208 | East Australia | 2.033727 | -0.17161 |
| C171669 | East Australia | 2.105987 | -0.15068 |
| F78082 | East Australia | 2.068866 | -0.24301 |
| F78281a | East Australia | 2.233548 | -0.12304 |
| F78283b | East Australia | 1.930682 | -0.27489 |
| F80442a | East Australia | 2.057868 | -0.14028 |
| F80442b | East Australia | 2.301519 | -0.14622 |
| F80446 | East Australia | 1.697702 | -0.12759 |
| F85370a | East Australia | 2.169133 | -0.19117 |
| F200320 | East Australia | 2.247057 | -0.17498 |
| F200319 | East Australia | 2.738278 | -0.17541 |
| F200318a | East Australia | 2.211669 | -0.14806 |
| F200318c | East Australia | 1.685601 | -0.10294 |
| F78283c | East Australia | 2.07602 | -0.26773 |
| F180704 | Tasmania | 2.186593 | -0.12979 |
| F180701 | Tasmania | 2.216311 | -0.01233 |
| F180705 | Tasmania | 2.93645 | 0.054955 |
| F200334 | Western Australia | 2.967886 | -0.16133 |
| F160320 | Western Australia | 2.371038 | -0.08675 |
| F160321 | Western Australia | 2.548854 | -0.23915 |
| F160325 | Western Australia | 2.2995 | -0.21815 |
| F80447 | Western Australia | 2.241005 | -0.05659 |
| F200327 | Western Australia | 2.365949 | -0.21258 |
| F200331 | Western Australia | 2.799516 | -0.14644 |
| F160302 | Western Australia | 2.37516 | -0.14816 |

Table S12: Eigenvalues and principal component (PC) variance contribution outputs from female octopod multivariate analysis.

|  | **Eigenvalue** | **PC variance contribution (%)** |
| --- | --- | --- |
| 1 | 2.007419303 | 70.7 |
| 2 | 0.832428114 | 29.3 |
| Total | 2.839847417 | 100 |

Table S13: Timing of divergence estimates (Tamura-Nei genetic distance) for *Octopus tetricus* (East Australia and New Zealand) and *O.* cf. *tetricus* (Western Australia).

|  | *Octopus tetricus* | *Octopus* cf. *tetricus* | Divergence (million years) | - | + |
| --- | --- | --- | --- | --- | --- |
| *Octopus tetricus* | 0.0021 | 0.0336 | 4.4 | 3.2 | 6.9 |
| *Octopus* cf. *tetricus* | 0.0336 | 0.0018 |  |  |  |

Table S14: Timing of divergence estimates (Tamura-Nei genetic distance) for the Australasian tetricus complex and Japanese/Chinese representatives of the *Octopus vulgaris* group.

|  | Japan/China | Australasia | Divergence (million years) | - | + |
| --- | --- | --- | --- | --- | --- |
| Japan/China | 0.0016 | 0.0570 | 7.4 | 5.4 | 11.6 |
| Australasia | 0.0570 | 0.0019 |  |  |  |
